# Supplementary material for: Integrated epidemiological and molecular data inform the relationship between precancer and cancer states of esophageal adenocarcinoma
Source: Nat Med. 2026 Apr 16;32(5):1805–16. doi: 10.1038/s41591-026-04331-8 (PMC13190344; doi:10.1038/s41591-026-04331-8)
Supplement: Supplementary file 1 — Supplementary Table 1. Exact statistical values for each pair of comparisons of esophageal adenocarcinoma (EAC) phenotypes that are presented in Fig. 5a. Supplementary Table 2. List of variables included for the analysis from the OCCAMS study. Bold indicates variables that were selected for further analysis using the selection process. Supplementary Fig. 1. Subgroup analysis of epigenetic and Barrett’s esophagus (BE)-relevant genomic phenotypes comparing combinations of endoscopic (macro) and pathological (micro) BE diagnosis. Supplementary Fig. 2. Subgroup analysis of EAC driver gene variations comparing combinations of endoscopic (macro) and pathological (micro) BE diagnosis. Supplementary Fig. 3. Schematic for deriving the heartburn variable using a combination of reflux-related variables. [file 41591_2026_4331_MOESM1_ESM.pdf]

nature medicine

Supplementary information https://doi.org/10.1038/s41591-026-04331-8

Integrated epidemiological and molecular data inform the relationship between precancer and cancer states of esophageal adenocarcinoma

In the format provided by the authors and unedited

Supplemental Information for manuscript entitled, "Integrated epidemiological and molecular data inform the relationship between precancer and cancer states of oesophageal adenocarcinoma"

Authors: S. A. Zamani, L. Wu, E. L. Black, A. Bartram, A. W. T. Ng, M. Secrier, D., J. D Perelman, A. Ustaoglu, [1] E. Ococks, D. Jacobson, G. Devonshire, N. Grehan, B. Nutzinger, A. Freeman, A. Miremadi, M., O'Donovan, A. M. Frankell, S. Killcoyne, OCCAMS Consortium, H. G. Coleman, and R. C. Fitzgerald.

Table of Contents

***Supplementary Table 1. Exact statistical values for each pairs of comparison of oesophageal adenocarcinoma phenotypes that presented in Figure 5A.*......................... 2**

***Supplementary Table 2. List of variables included for the analysis from the OCCAMS study. Bold indicates variables which were selected for further analysis using the selection process.*.. 3**

***Supplementary Figure 1. Subgroup analysis of epigenetic and BE-relevant genomic phenotypes comparing combinations of endoscopic (macro) and pathological (micro) Barrett's oesophagus (BE) diagnosis.*.. 5**

***Supplementary Figure 2. Subgroup analysis of oesophageal adenocarcinoma (EAC) driver gene variations comparing combinations of endoscopic (macro) and pathological (micro) Barrett's oesophagus (BE) diagnosis.*.. 6**

***Supplementary Figure 3. Schematic for deriving the heartburn variable using a combination of reflux-related variables.*... 7**

***Source data legends*.. 8**

Supplementary Table 1. Exact statistical values for each pairs of comparison of oesophageal adenocarcinoma phenotypes that presented in Figure 5A.

group1	group2	chisq_stat	df	p_value	p_adj_fdr	p_adj_bonferroni
Full_BE (?)	Full_BE+ve	46.74	3	0.00	0.00	0.00
Full_BE (?)	Full_BE-ve	12.27	3	0.01	0.01	0.10
Full_BE (?)	Surveillance_BE (?)	28.95	3	0.00	0.00	0.00
Full_BE (?)	Surveillance_BE+ve	145.88	3	0.00	0.00	0.00
Full_BE (?)	Surveillance_BE-ve	2.15	3	0.54	0.58	1.00
Full_BE+ve	Full_BE-ve	97.27	3	0.00	0.00	0.00
Full_BE+ve	Surveillance_BE (?)	8.79	3	0.03	0.05	0.48
Full_BE+ve	Surveillance_BE+ve	68.77	3	0.00	0.00	0.00
Full_BE+ve	Surveillance_BE-ve	0.15	3	0.99	0.99	1.00
Full_BE-ve	Surveillance_BE (?)	48.84	3	0.00	0.00	0.00
Full_BE-ve	Surveillance_BE+ve	207.80	3	0.00	0.00	0.00
Full_BE-ve	Surveillance_BE-ve	5.21	3	0.16	0.20	1.00
Surveillance_BE (?)	Surveillance_BE+ve	5.71	3	0.13	0.17	1.00
Surveillance_BE (?)	Surveillance_BE-ve	2.85	3	0.42	0.48	1.00
Surveillance_BE+ve	Surveillance_BE-ve	12.33	3	0.01	0.01	0.09

Supplementary Table 2. List of variables included for the analysis from the OCCAMS study. Bold indicates variables which were selected for further analysis using the selection process.

Domain	Variable
**Demographics**	**Age at diagnosis**
	**Sex**
	Ethnicity
**Risk factor exposures**	**BMI at baseline; *kg/m²***
	BMI five years prior to diagnosis; *kg/m²*
	BMI difference (prior to baseline); *kg/m²*
	**Cigarette smoking status**
	Number of cigarettes smoked per day
	Years of smoking cigarettes
	Number of pack-years of smoking
	Heavy alcohol drinking status
	Units of alcohol intake per week
**Anti-inflammatory medications**	Aspirin use status
	Years of aspirin use
	NSAID use status
	Years of NSAID use
	**Any use of aspirin or NSAID**
**Reflux symptoms & acid suppressant medications**	Frequency of reflux symptoms
	Duration since reflux symptoms began
	Currently taking acid suppressant medications
	Currently symptomatic for reflux while on acid-suppressants
	PPI medication use status
	Years of PPI medication use
	OTC acid suppressant medication use status
	Years of OTC medication use status
	H2RA medication use
	Years of H2RA medication use
	Use of any acid suppressant/reducing medications status
	**Derived heartburn symptom status**
**Clinical factors**	Tumor length, *cm*
	Tumor growth (T stage pre-op to T stage post-op)
	**TNM**

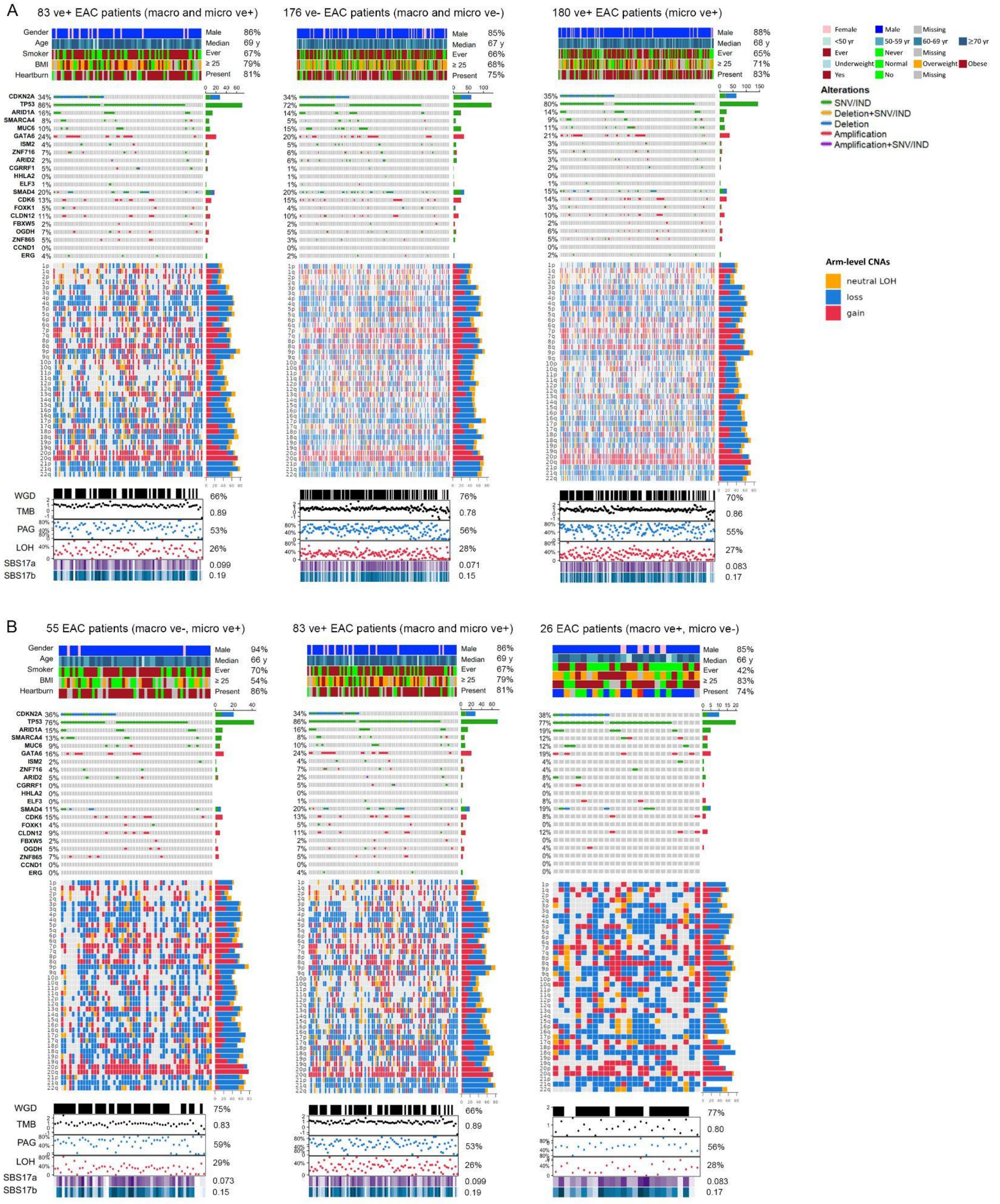
A
83 ve+ EAC patients (macro and micro ve+)
176 ve- EAC patients (macro and micro ve-)
180 ve+ EAC patients (micro ve+)
B
55 EAC patients (macro ve-, micro ve+)
83 ve+ EAC patients (macro and micro ve+)
26 EAC patients (macro ve+, micro ve-)
Gender
Age
Smoker
BMI
Heartburn
Female
Male
Missing
<50 yr
50-59 yr
60-69 yr
≥70 yr
Ever
Never
Underweight
Normal
Overweight
Obese
Yes
No
Alterations
SNV/IND
Deletion+SNV/IND
Deletion
Amplification
Amplification+SNV/IND
Arm-level CNAs
neutral LOH
loss
gain
WGD
TMB
PAG
LOH
SBS17a
SBS17b

Supplementary Figure 1. Subgroup analysis of epigenetic and BE-relevant genomic phenotypes comparing combinations of endoscopic (macro) and pathological (micro) Barrett's oesophagus (BE) diagnosis.

No statistically significant differences were found across any subgroup comparisons.

A1 vs. A2 (Most stringent comparison, same criteria as Table R1): Comparison of cases with both endoscopic and pathological evidence of BE (n=83) versus cases with no evidence by either method (n=176).

A3 vs. A2 (Micro +ve vs. fully negative, same criteria as Table R2): Comparison of all pathology-positive cases regardless of endoscopy (n=180) versus fully negative cases (n=176).

B1 vs. B2 (Internal comparison within pathology/microscopically positive cases, same criteria as Table R3): 53 cases with pathology-positive but endoscopy-negative findings versus 83 cases positive by both methods.

B3 vs. B2 (Internal comparison within endoscopy/macroscopically positive cases, same criteria as Table R4): 26 cases with endoscopy-positive but pathology-negative findings versus 83 cases positive by both methods.

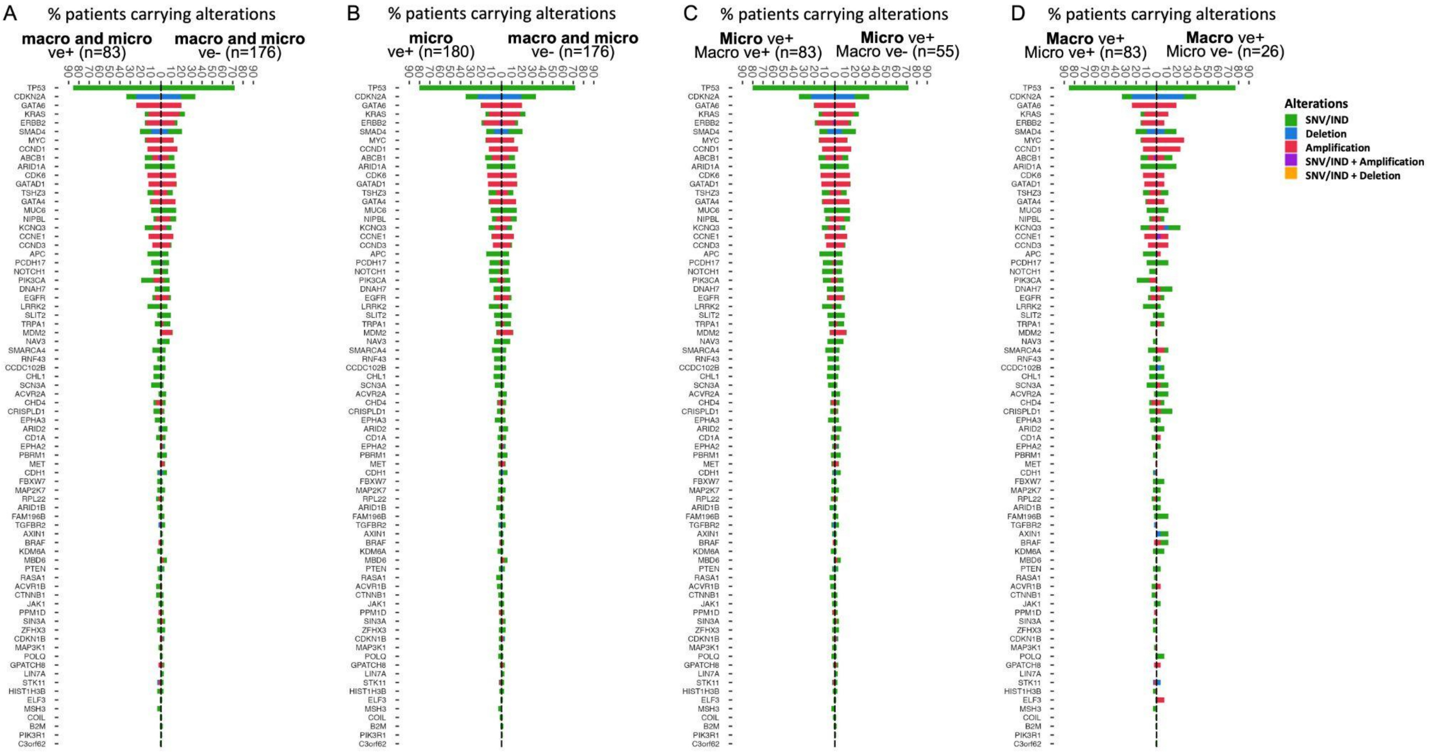

Supplementary Figure 2. Subgroup analysis of oesophageal adenocarcinoma (EAC) driver gene variations comparing combinations of endoscopic (macro) and pathological (micro) Barrett's oesophagus (BE) diagnosis.

Overall no significant differences were found. Panels A–D correspond respectively to the grouping criteria in Table R1-R4: (A) the most stringent comparison, (B) micro-positive vs. fully negative, (C) internal comparison within pathology-positive cases, and (D) internal comparison within endoscopy-positive cases.

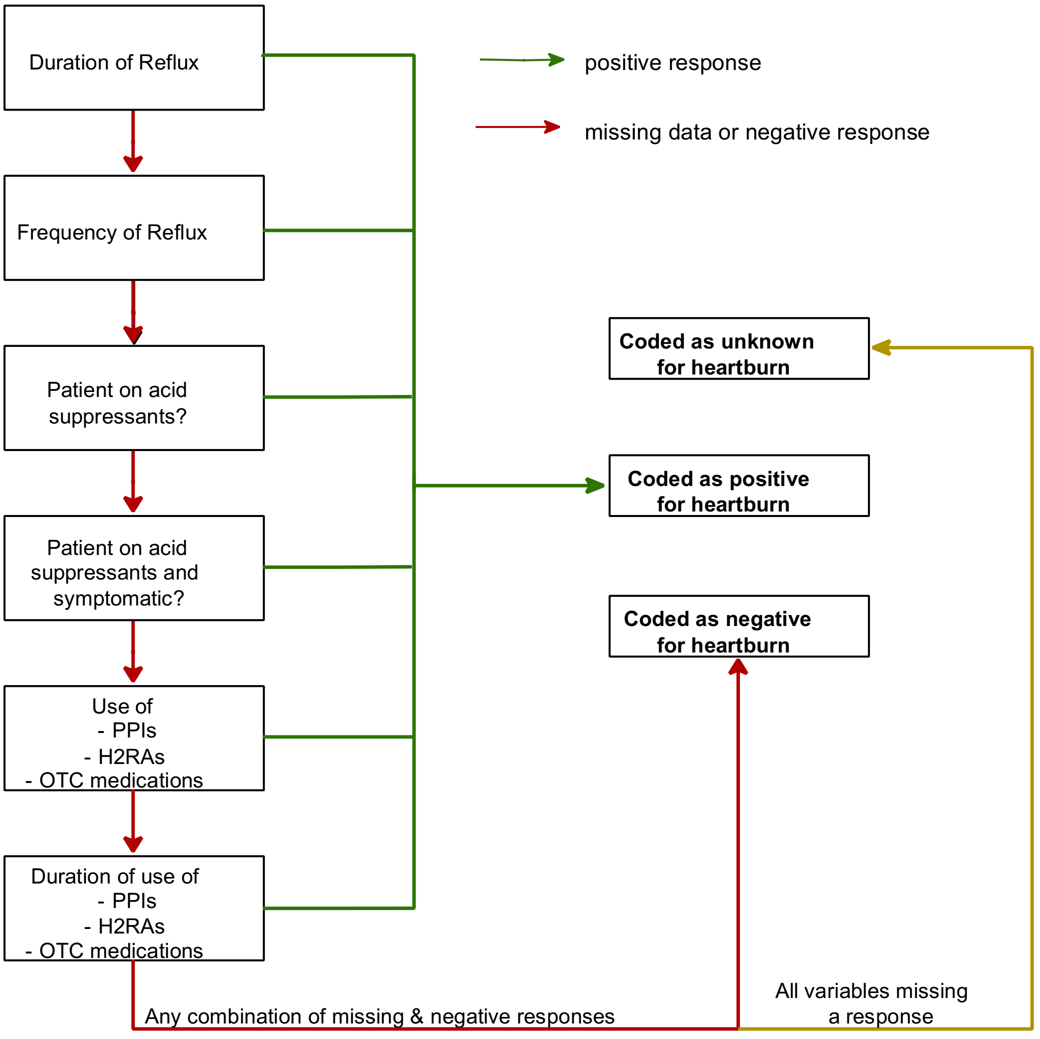

Supplementary Figure 3. Schematic for deriving the heartburn variable using a combination of reflux-related variables.

Abbreviations: PPIs, proton pump inhibitors; H2RAs, histamine H2-receptor antagonists; OTC, over the-counter.

Source data legends

Source data Figure 2. Comparison of Barrett's driver gene mutations and arm-level copy number alterations between Barrett's oesophagus (BE) and oesophageal adenocarcinoma (EAC), and between BE positive and negative phenotypes of EAC.

Source data Figure 3A. Comparison of oesophageal adenocarcinoma (EAC) driver gene mutations between Barrett's oesophagus (BE) positive and negative phenotypes of EAC.

Source data Figure 4A. Phylogenetic trees for all patients, ordered by clusters. Phylogenetic trees were generated from multi-regional whole exome sequencing (WES) cohort. The trees are displayed according to their BE associated phenotype and evolutionary clusters. The trunks typically display more driver genes in more advanced cases, but the driver gene events and overall tree shape are very similar between phenotypes.

Source data Figure 4C. Comparison of clonal-to-subclonal ratios for driver genes in the multiregional whole-exome sequencing cohort.

Source data Figure 5B. The raw images of spatial transcriptomics data.
