# Supplementary material for: Integrated epidemiological and molecular data inform the relationship between precancer and cancer states of esophageal adenocarcinoma
Source: Nat Med. 2026 Apr 16;32(5):1805–16. doi: 10.1038/s41591-026-04331-8 (PMC13190344; doi:10.1038/s41591-026-04331-8)
Supplement: Supplementary file 2 — Reporting Summary [file 41591_2026_4331_MOESM2_ESM.pdf]

nature portfolio

Corresponding author(s): Rebecca C Fitzgerald

Last updated by author(s): 05/02/2026

Reporting Summary

Nature Portfolio wishes to improve the reproducibility of the work that we publish. This form provides structure for consistency and transparency in reporting. For further information on Nature Portfolio policies, see our Editorial Policies and the Editorial Policy Checklist.

Statistics

For all statistical analyses, confirm that the following items are present in the figure legend, table legend, main text, or Methods section.

n/a	Confirmed	
☐	☒	The exact sample size (*n*) for each experimental group/condition, given as a discrete number and unit of measurement
☐	☒	A statement on whether measurements were taken from distinct samples or whether the same sample was measured repeatedly
☐	☒	The statistical test(s) used AND whether they are one- or two-sided<br>*Only common tests should be described solely by name; describe more complex techniques in the Methods section.*
☐	☒	A description of all covariates tested
☐	☒	A description of any assumptions or corrections, such as tests of normality and adjustment for multiple comparisons
☐	☒	A full description of the statistical parameters including central tendency (e.g. means) or other basic estimates (e.g. regression coefficient) AND variation (e.g. standard deviation) or associated estimates of uncertainty (e.g. confidence intervals)
☐	☒	For null hypothesis testing, the test statistic (e.g. *F*, *t*, *r*) with confidence intervals, effect sizes, degrees of freedom and *P* value noted<br>*Give P values as exact values whenever suitable.*
☒	☐	For Bayesian analysis, information on the choice of priors and Markov chain Monte Carlo settings
☐	☒	For hierarchical and complex designs, identification of the appropriate level for tests and full reporting of outcomes
☒	☐	Estimates of effect sizes (e.g. Cohen's *d*, Pearson's *r*), indicating how they were calculated

Our web collection on statistics for biologists contains articles on many of the points above.

Software and code

Policy information about availability of computer code

Data collection	n/a
Data analysis	https://github.com/fitzgerald-lab/EAC-Phenotypes - analysis pipeline, packages and versions are detailed here

For manuscripts utilizing custom algorithms or software that are central to the research but not yet described in published literature, software must be made available to editors and reviewers. We strongly encourage code deposition in a community repository (e.g. GitHub). See the Nature Portfolio guidelines for submitting code & software for further information.

Data

Policy information about availability of data

All manuscripts must include a data availability statement. This statement should provide the following information, where applicable:

- Accession codes, unique identifiers, or web links for publicly available datasets
- A description of any restrictions on data availability
- For clinical datasets or third party data, please ensure that the statement adheres to our policy

The data that support the findings of this work are available as follows: The data that support the findings of this work are available as follows: WGS data for samples sequenced by Illumina or the CRUK Cambridge Institute are available at the European Genome-phenome Archive (EGA) under accession number EGAD00001011191 . WGS data for samples sequenced by the Wellcome Sanger Institute are available at the EGA under accession number EGAD00001006083.

WGS data for samples sequenced by Genomics England are available in the Genomics England's National Genomic Research Library https://www.genomicsengland.co.uk/research/data.

Research involving human participants, their data, or biological material

Policy information about studies with human participants or human data. See also policy information about sex, gender (identity/presentation), and sexual orientation and race, ethnicity and racism.

Reporting on sex and gender	Gender was determined based on self-reported questionnaire data
Reporting on race, ethnicity, or other socially relevant groupings	according to policy
Population characteristics	This is described in Table 1 and Extended Data Tables 1, 2 and 3
Recruitment	Prospective, individual informed consent. Details are given in the manuscript "selection of cases"
Ethics oversight	East of England Ethics Committees

Note that full information on the approval of the study protocol must also be provided in the manuscript.

Field-specific reporting

Please select the one below that is the best fit for your research. If you are not sure, read the appropriate sections before making your selection.

☒ Life sciences ☐ Behavioural & social sciences ☐ Ecological, evolutionary & environmental sciences

For a reference copy of the document with all sections, see nature.com/documents/nr-reporting-summary-flat.pdf

Life sciences study design

All studies must disclose on these points even when the disclosure is negative.

Sample size	No statistical method was used to predetermine sample size. However, this study represents the largest clinical and genomic cohort to date, with the next largest cohorts having 54% fewer patients with clinical, and 31% and 49% fewer EAC and BE genomically sequenced patients, respectively.
Data exclusions	We removed samples/data of poor quality. Details are given in the manuscript
Replication	For the epidemiological and genomic data analysis, we used different methods (technical replicates) to provide evidence from different aspects. For staining, biological replicates were done with different markers.
Randomization	The experiments were not randomized. Randomization is not suitable as this is a real world dataset.
Blinding	The pathologist-investigators were blinded to biopsy results, the risk factor data (heartburn, smoking, and BMI). Other investigators were not blinded to the independent variables and phenotype outcome.

Reporting for specific materials, systems and methods

We require information from authors about some types of materials, experimental systems and methods used in many studies. Here, indicate whether each material, system or method listed is relevant to your study. If you are not sure if a list item applies to your research, read the appropriate section before selecting a response.

Materials & experimental systems

n/a	Involved in the study	
☐	☒	Antibodies
☒	☐	Eukaryotic cell lines
☒	☐	Palaeontology and archaeology
☒	☐	Animals and other organisms
☐	☒	Clinical data
☒	☐	Dual use research of concern
☒	☐	Plants

Methods

n/a	Involved in the study	
☒	☐	ChIP-seq
☒	☐	Flow cytometry
☒	☐	MRI-based neuroimaging

Antibodies

Antibodies used	We used TFF3 antibody from Invitrogen (0.26 ug/ml), and REG4 antibody from Abcam #ab255820 (0.26ug/ml).
Validation	According to the manufacturer, the TFF3 antibody (#MA5-42854, clone 7X6O2, RRID AB_2911995) was validated by IHC in HEK293 cells, and human colon carcinoma tissue (Thermofisher scientific datasheet), with published images on the product page. The REG4 antibody (#ab255820, clone EPR22810-327) was validated by the manufacturer on human colon tissue using IHC at 1/2000 (0.262 µg/ml) and positive staining observed, with published images on product page (Abcam datasheet). Specificity was confirmed by absence of signal in squamous epithelium of normal oesophageal biopsies, and strong expression in human colon tissue for both TFF3 and REG4 antibodies.

Clinical data

Policy information about clinical studies

All manuscripts should comply with the ICMJE guidelines for publication of clinical research and a completed CONSORT checklist must be included with all submissions.

Clinical trial registration	UKCRNID-8880, REC 07/H0305/52 and 10/H0305/1
Study protocol	OCCAMS study protocol available from study coordinators on request
Data collection	Prospective
Outcomes	epidemiological and genomic profiling correlated with presence or absence of Barretts Oesophagus

Plants

Seed stocks	*Report on the source of all seed stocks or other plant material used. If applicable, state the seed stock centre and catalogue number. If plant specimens were collected from the field, describe the collection location, date and sampling procedures.*
Novel plant genotypes	*Describe the methods by which all novel plant genotypes were produced. This includes those generated by transgenic approaches, gene editing, chemical/radiation-based mutagenesis and hybridization. For transgenic lines, describe the transformation method, the number of independent lines analyzed and the generation upon which experiments were performed. For gene-edited lines, describe the editor used, the endogenous sequence targeted for editing, the targeting guide RNA sequence (if applicable) and how the editor was applied.*
Authentication	*Describe any authentication procedures for each seed stock used or novel genotype generated. Describe any experiments used to assess the effect of a mutation and, where applicable, how potential secondary effects (e.g. second site T-DNA insertions, mosiacism, off-target gene editing) were examined.*
