# Supplementary material for: Integrated epidemiological and molecular data inform the relationship between precancer and cancer states of esophageal adenocarcinoma
Source: Nat Med. 2026 Apr 16;32(5):1805–16. doi: 10.1038/s41591-026-04331-8 (PMC13190344; doi:10.1038/s41591-026-04331-8)
Supplement: Supplementary file 6 — Phylogenetic trees for all patients, ordered by clusters. Phylogenetic trees were generated from the multiregional whole exome sequencing cohort. [file 41591_2026_4331_MOESM6_ESM.pdf]

# Cluster 1

# BE+ve EAC

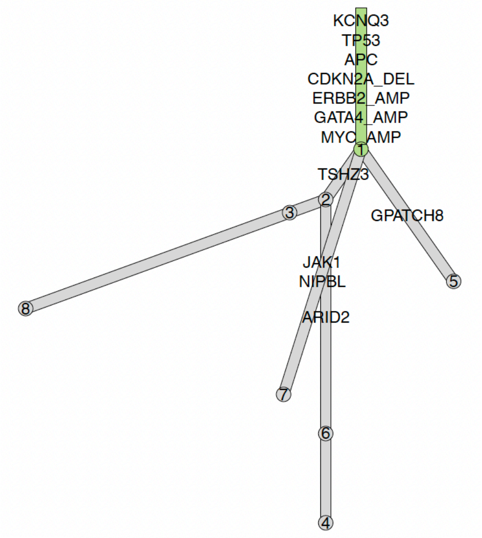


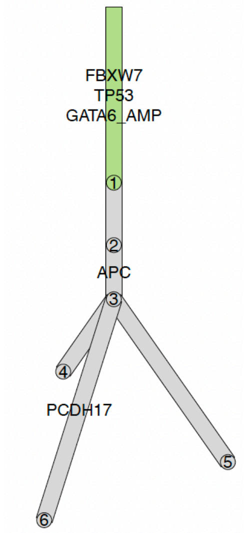


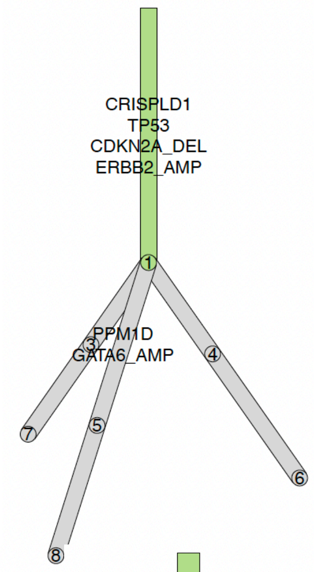


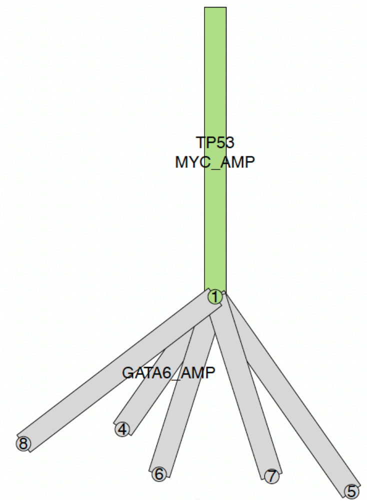


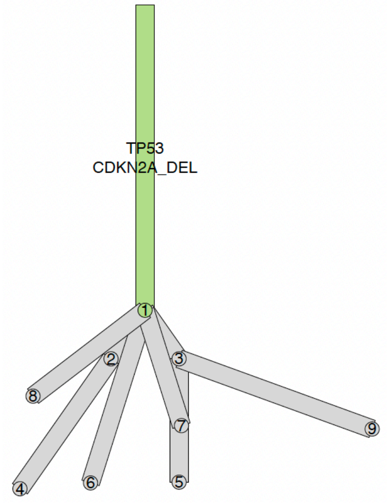


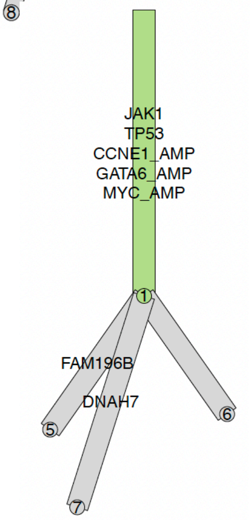


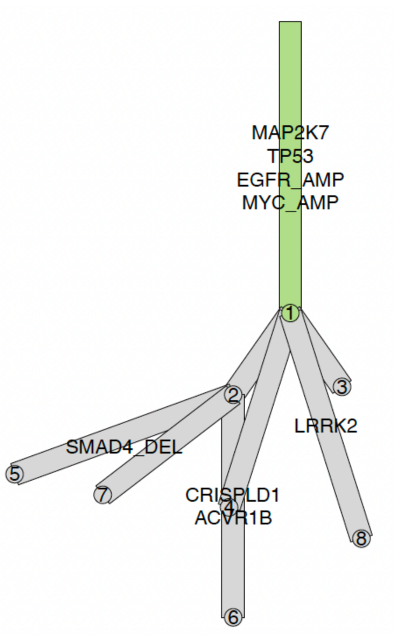


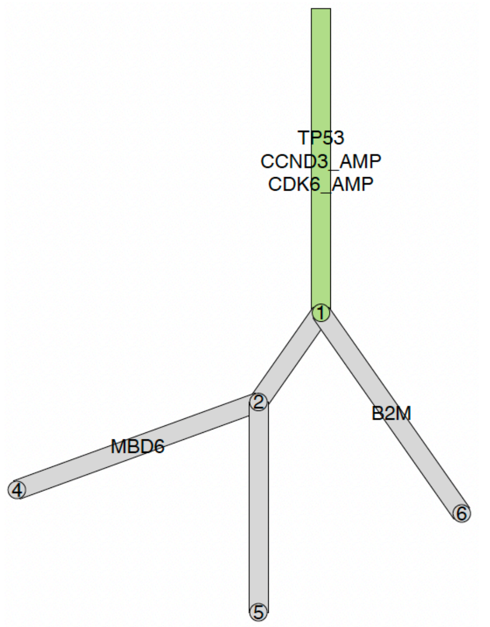


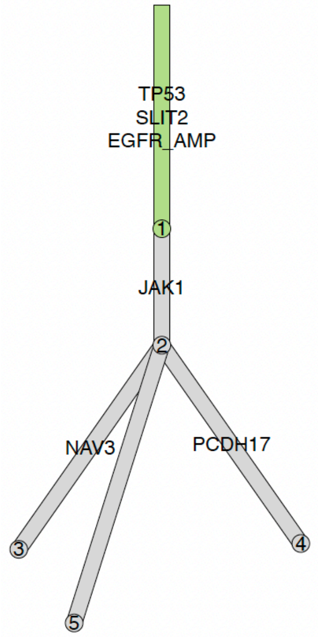

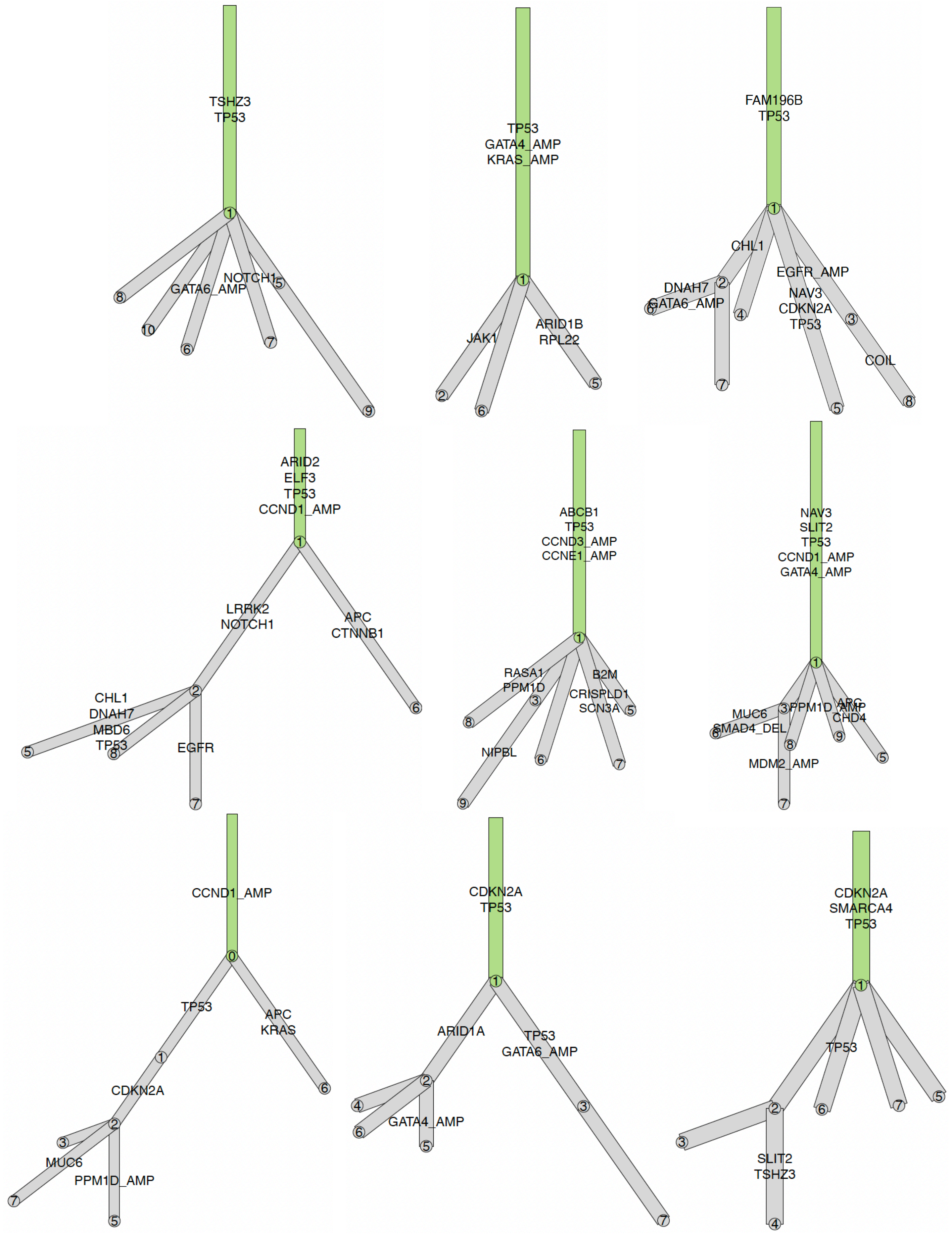

TSHZ3
TP53
GATA6_AMP
NOTCH1
TP53
GATA4_AMP
KRAS_AMP
JAK1
ARID1B
RPL22
FAM196B
TP53
CHL1
DNAH7
GATA6_AMP
EGFR_AMP
NAV3
CDKN2A
TP53
COIL
ARID2
ELF3
TP53
CCND1_AMP
LRRK2
NOTCH1
APC
CTNNB1
CHL1
DNAH7
MBD6
TP53
EGFR
ABCB1
TP53
CCND3_AMP
CCNE1_AMP
RASA1
PPM1D
B2M
CRISPLD1
SCN3A
NIPBL
NAV3
SLIT2
TP53
CCND1_AMP
GATA4_AMP
MUC6
SMAD4_DEL
PPM1D_AMP
APC
CHD4
MDM2_AMP
CCND1_AMP
TP53
APC
KRAS
CDKN2A
MUC6
PPM1D_AMP
CDKN2A
TP53
ARID1A
TP53
GATA6_AMP
GATA4_AMP
CDKN2A
SMARCA4
TP53
TP53
SLIT2
TSHZ3

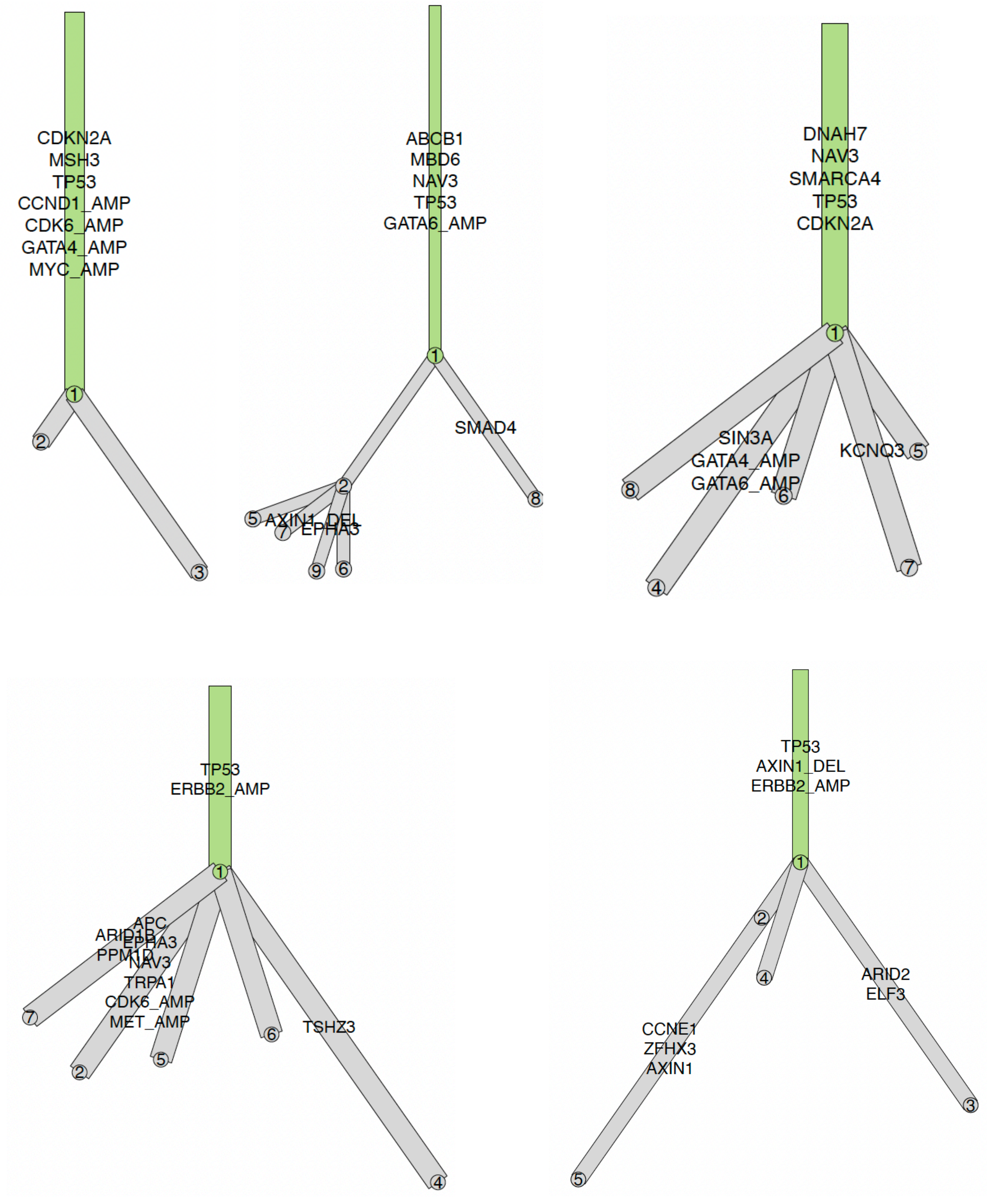
CDKN2A
MSH3
TP53
CCND1_AMP
CDK6_AMP
GATA4_AMP
MYC_AMP
ABCB1
MBD6
NAV3
TP53
GATA6_AMP
SMAD4
AXIN1_DEL
EPHA3
DNAH7
NAV3
SMARCA4
TP53
CDKN2A
SIN3A
GATA4_AMP
GATA6_AMP
KCNQ3
TP53
ERBB2_AMP
APC
ARID1B
EPHA3
PPM1D
NAV3
TRPA1
CDK6_AMP
MET_AMP
TSHZ3
TP53
AXIN1_DEL
ERBB2_AMP
ARID2
ELF3
CCNE1
ZFHX3
AXIN1

## Be-ve EAC

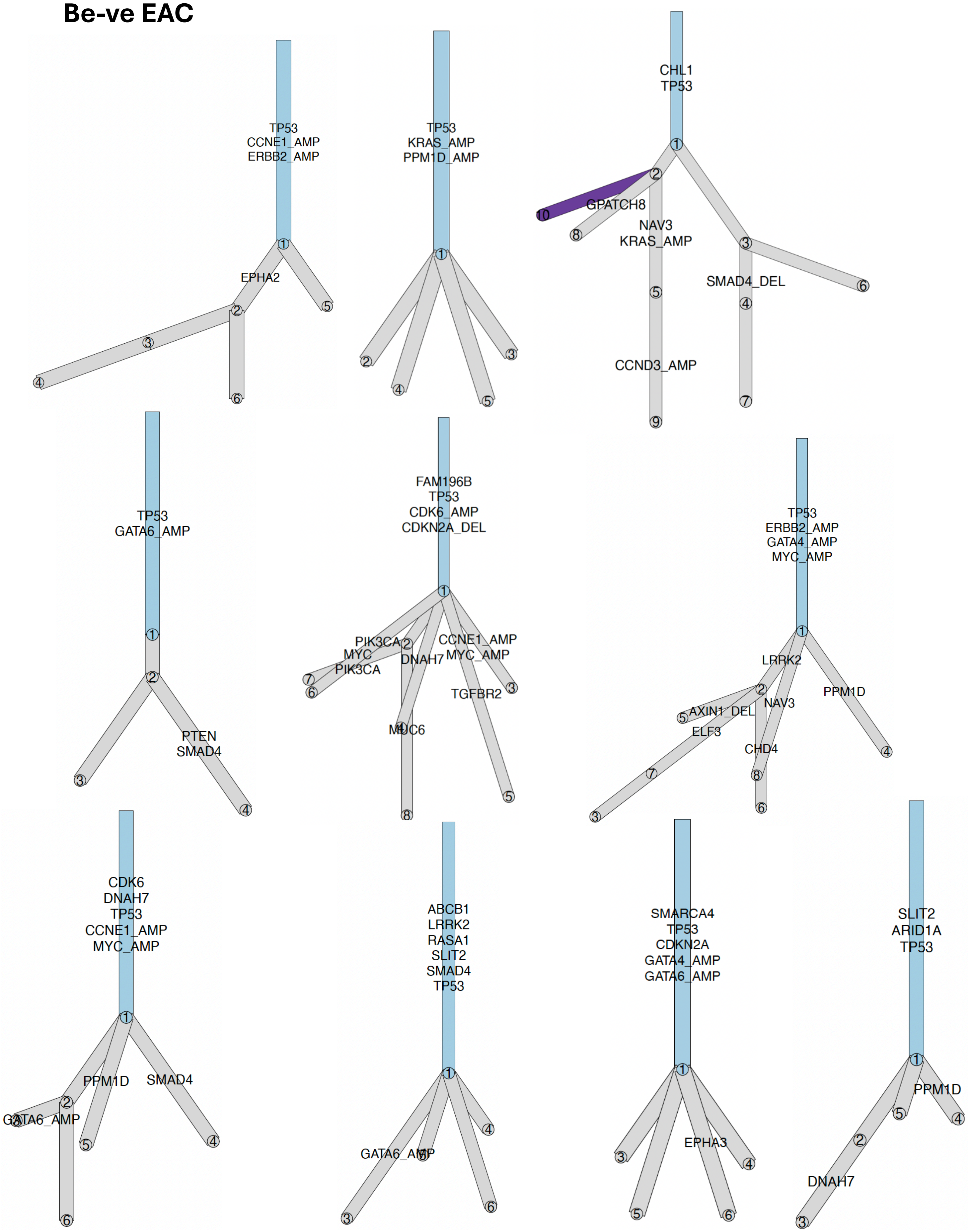

TP53
CCNE1_AMP
ERBB2_AMP
EPHA2
TP53
KRAS_AMP
PPM1D_AMP
CHL1
TP53
GPATCH8
NAV3
KRAS_AMP
SMAD4_DEL
CCND3_AMP
TP53
GATA6_AMP
PTEN
SMAD4
FAM196B
TP53
CDK6_AMP
CDKN2A_DEL
PIK3CA
MYC
PIK3CA
DNAH7
CCNE1_AMP
MYC_AMP
TGFBR2
MUC6
TP53
ERBB2_AMP
GATA4_AMP
MYC_AMP
LRRK2
AXIN1_DEL
ELF3
NAV3
CHD4
PPM1D
CDK6
DNAH7
TP53
CCNE1_AMP
MYC_AMP
PPM1D
SMAD4
GATA6_AMP
ABCB1
LRRK2
RASA1
SLIT2
SMAD4
TP53
GATA6_AMP
SMARCA4
TP53
CDKN2A
GATA4_AMP
GATA6_AMP
EPHA3
SLIT2
ARID1A
TP53
PPM1D
DNAH7

**Cluster 2**

**BE+ve EAC**

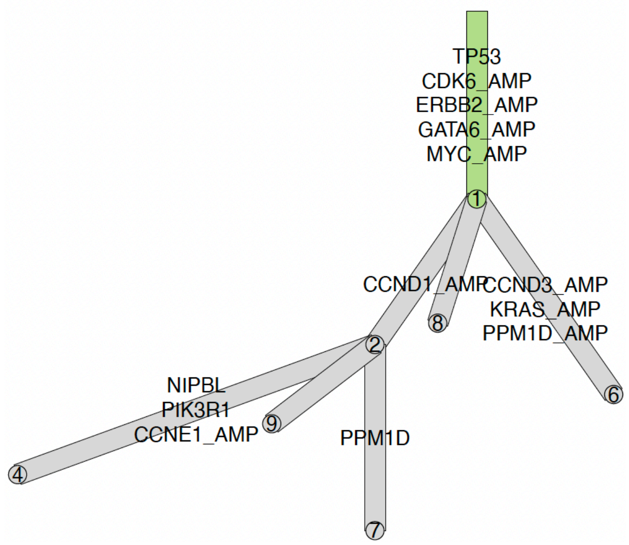


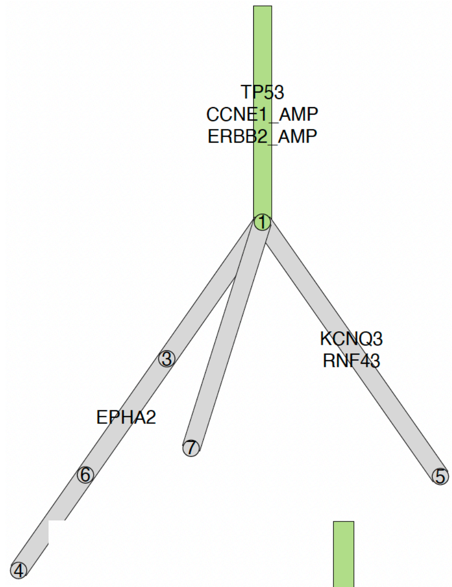


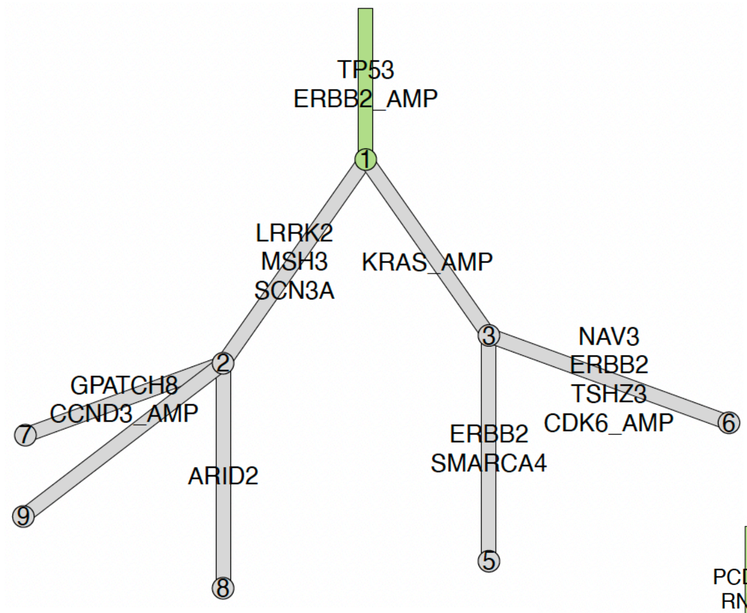


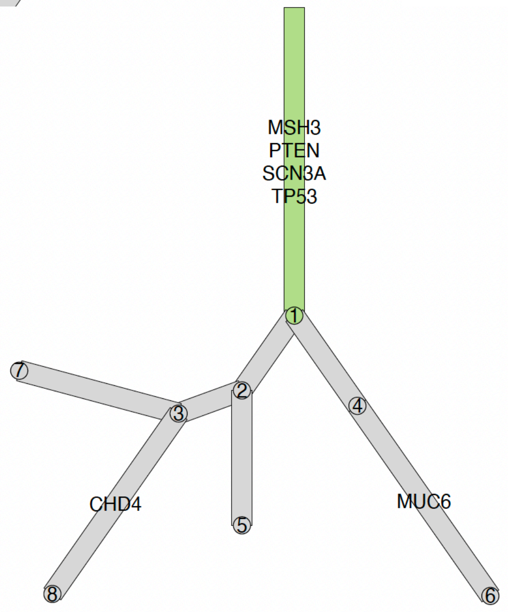


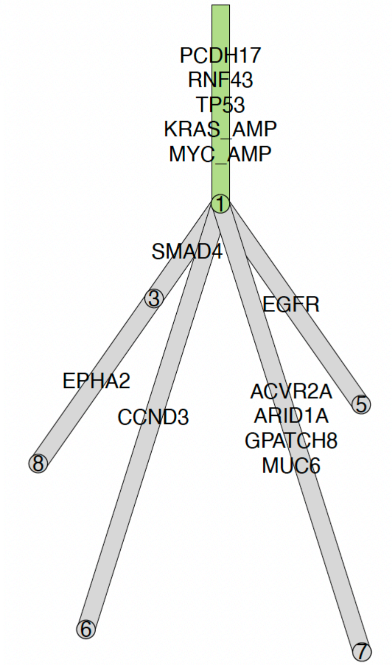

**BE-ve**

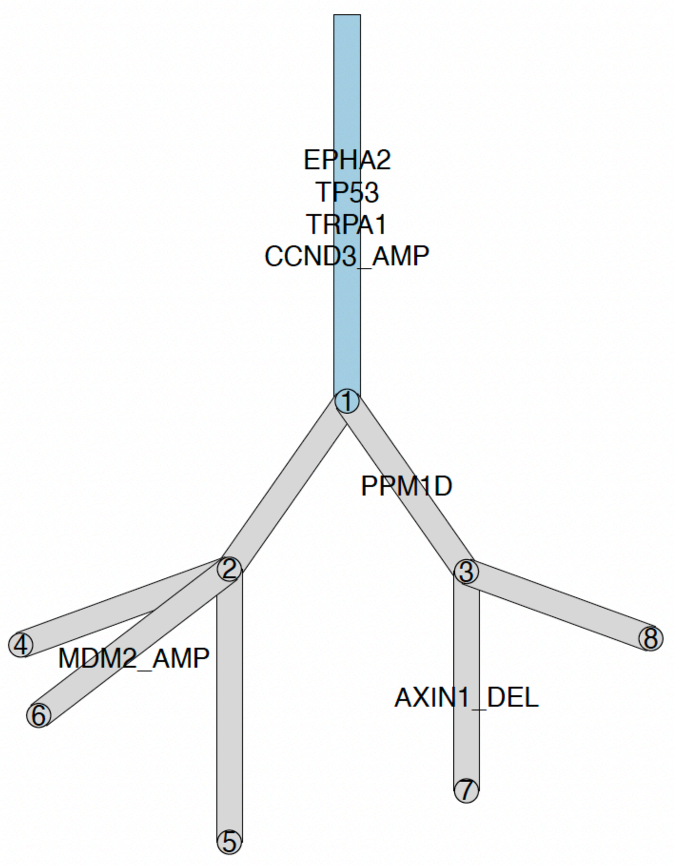
EPHA2
TP53
TRPA1
CCND3_AMP
PPM1D
MDM2_AMP
AXIN1_DEL
1
2
3
4
5
6
7
8

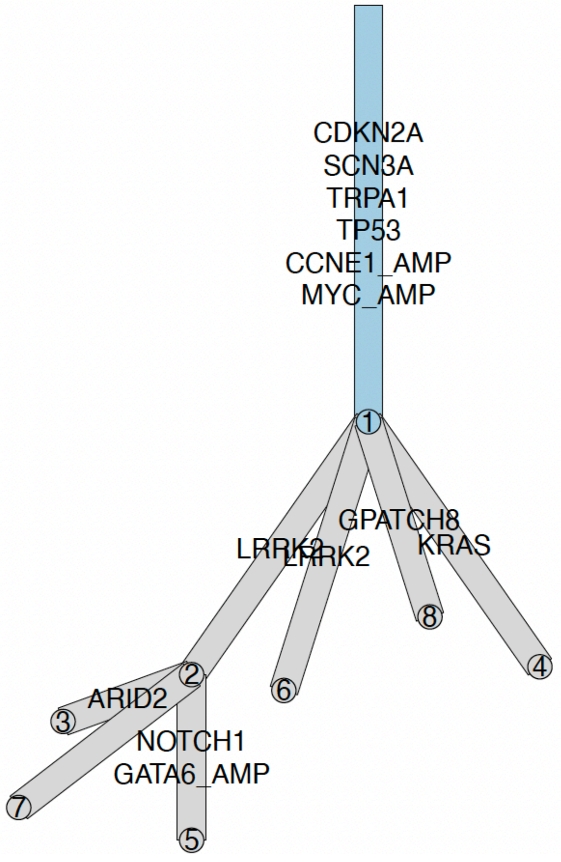
CDKN2A
SCN3A
TRPA1
TP53
CCNE1_AMP
MYC_AMP
GPATCH8
KRAS
LRRK2
LRRK2
ARID2
NOTCH1
GATA6_AMP
1
2
3
4
5
6
7
8

## Cluster 3

## BE+ve EAC

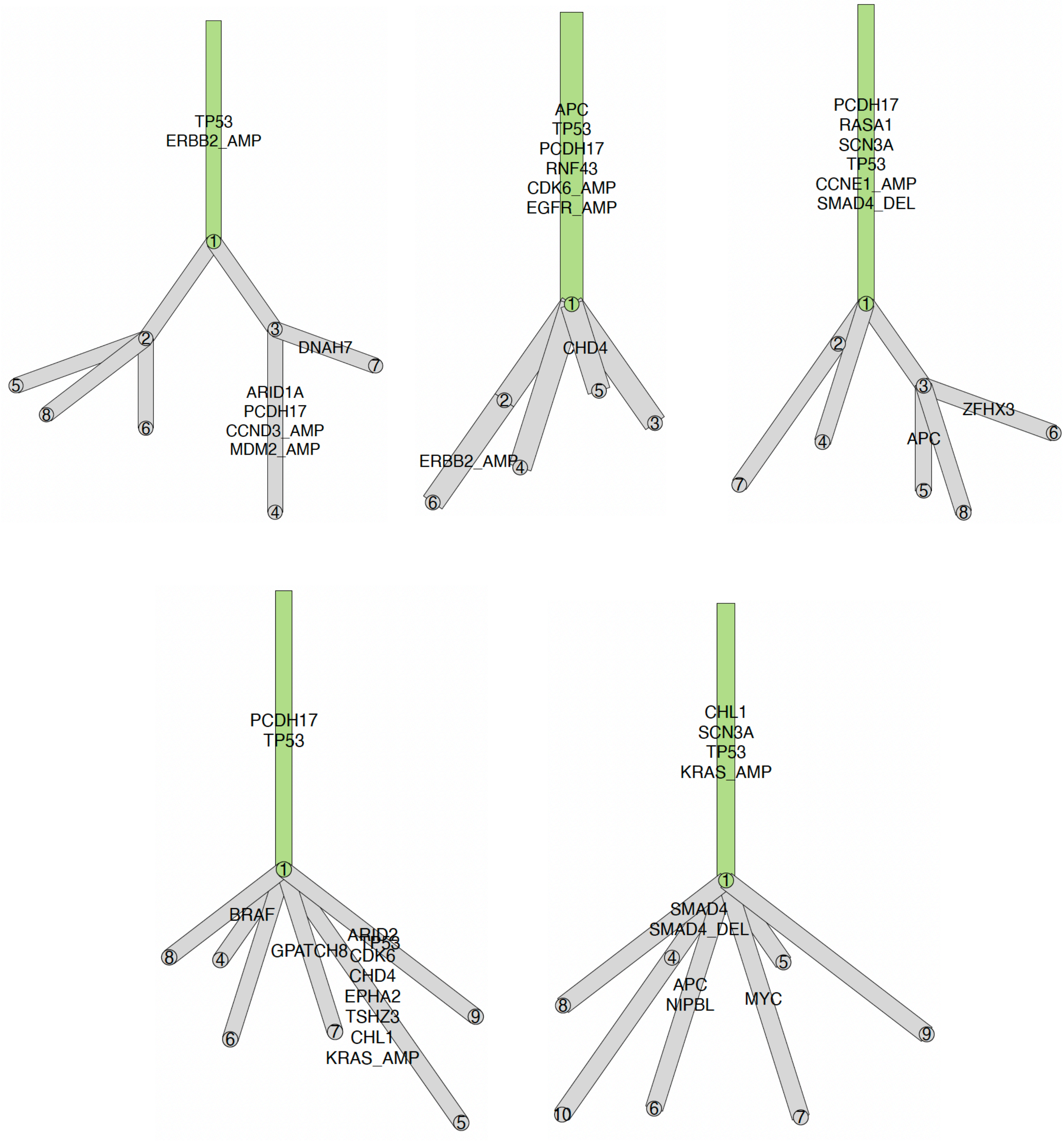

BE-ve EAC

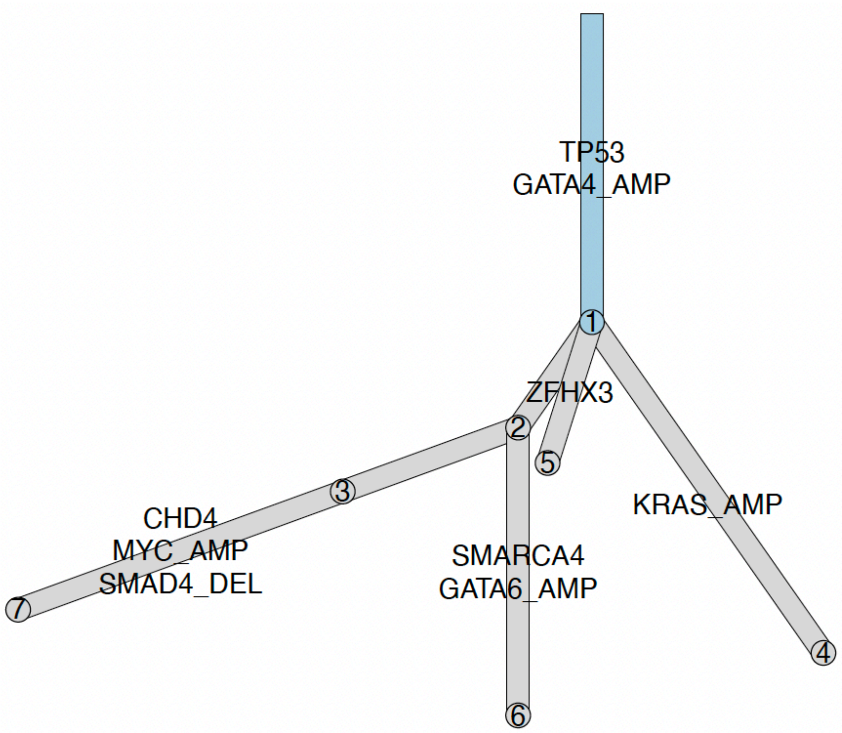

TP53
GATA4_AMP
1
ZFHX3
2
5
3
CHD4
MYC_AMP
SMAD4_DEL
7
KRAS_AMP
SMARCA4
GATA6_AMP
4
6

**Cluster 4**

**BE+ve EAC**

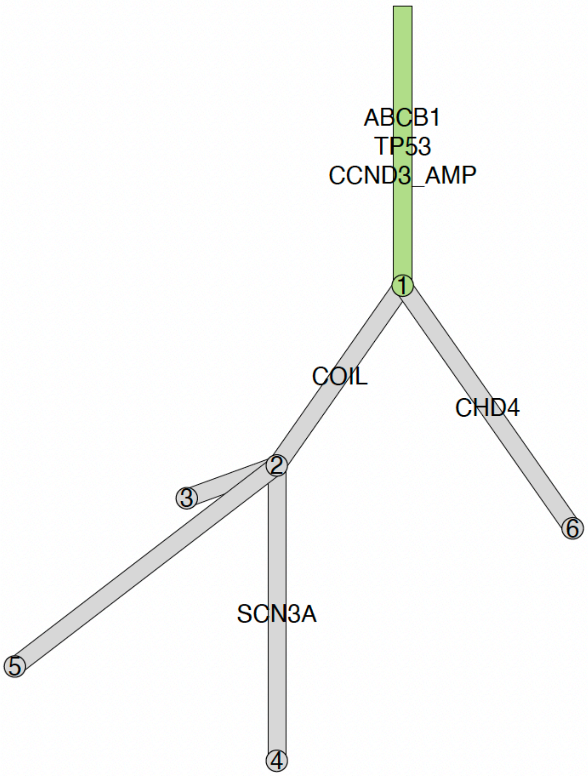


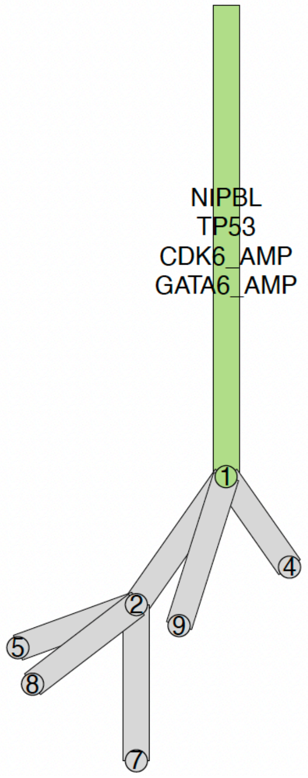


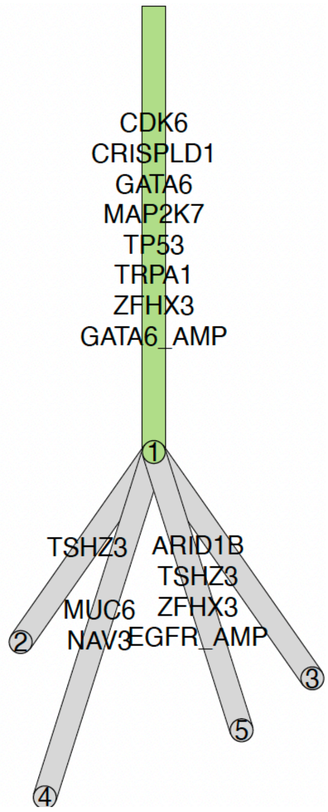


**BE-ve EAC**

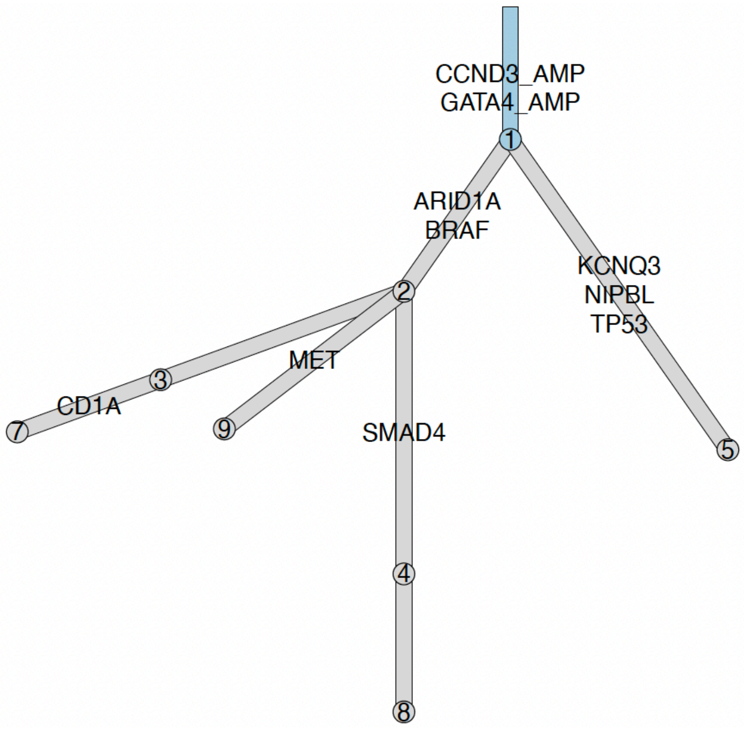


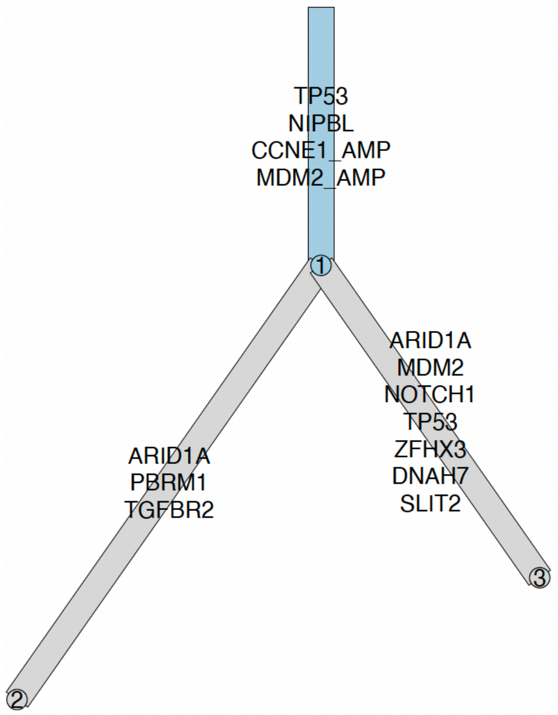

## Cluster 5

## BE+ve EAC

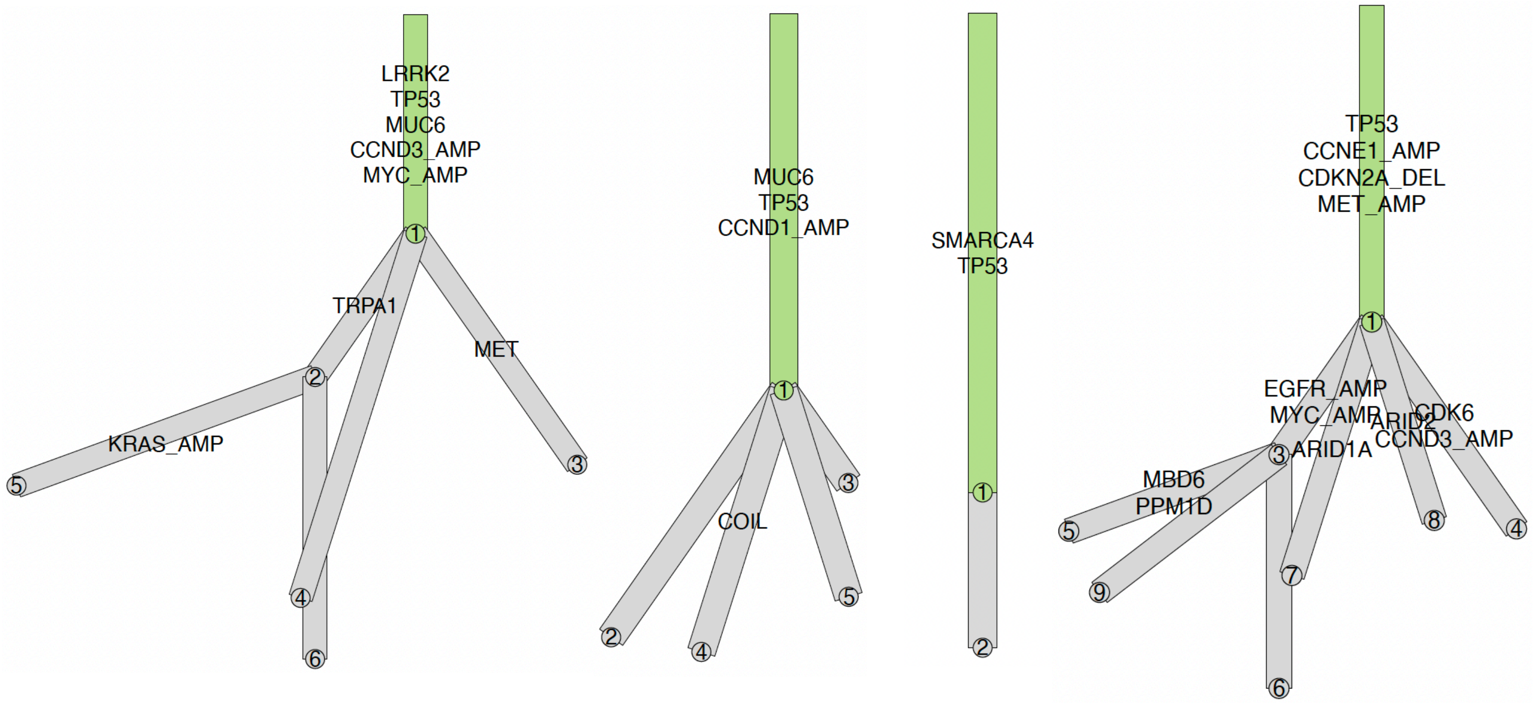


## BE-ve EAC

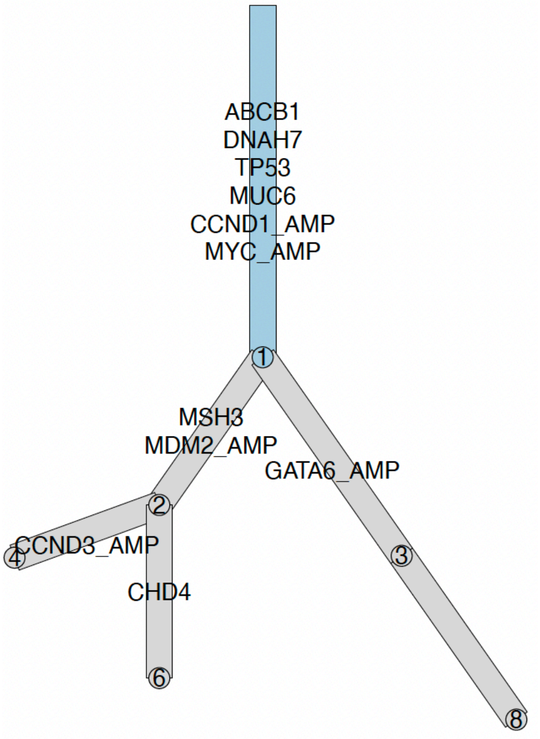

**Cluster 6**

**BE+ve EAC**

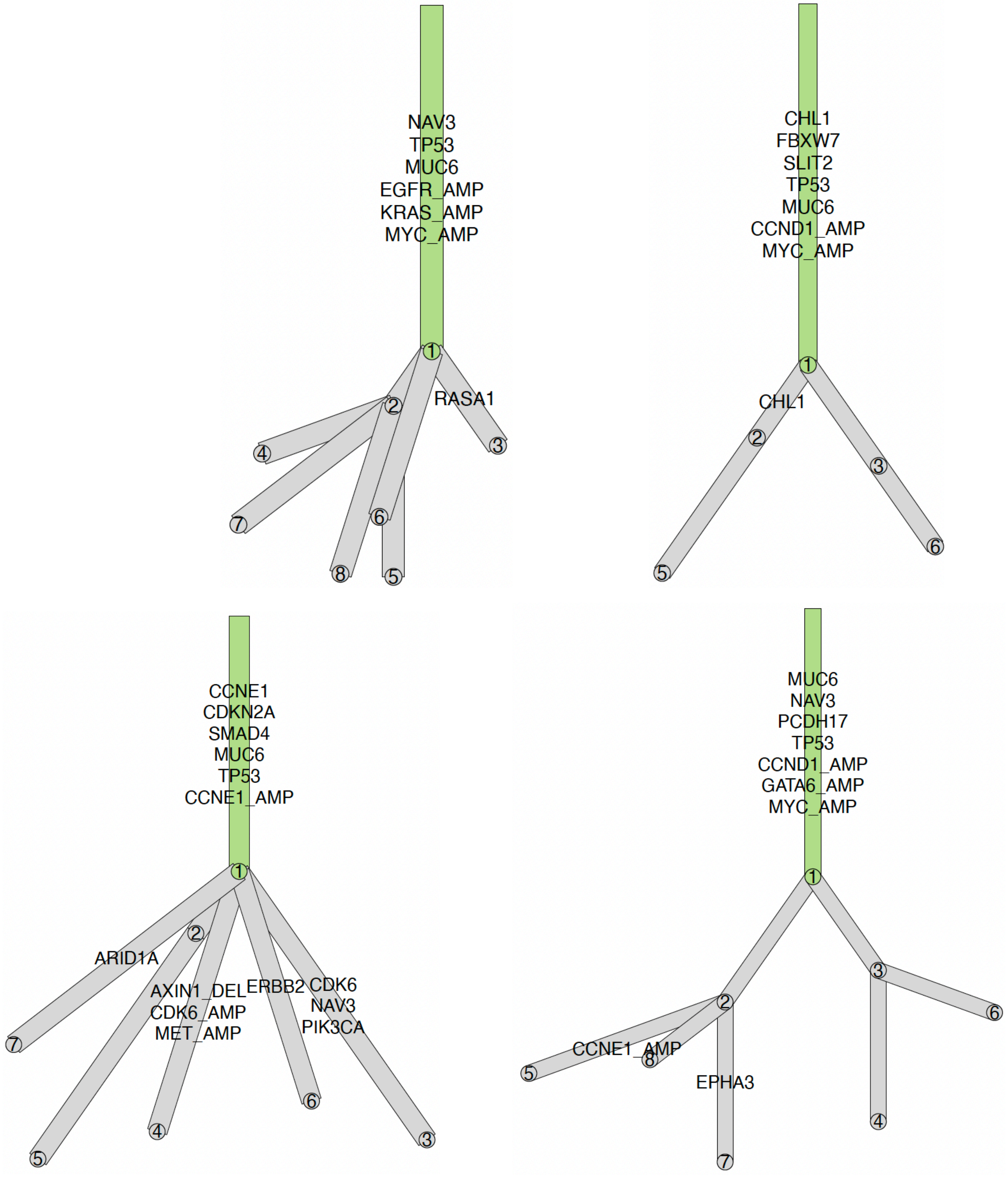

## BE-ve EAC

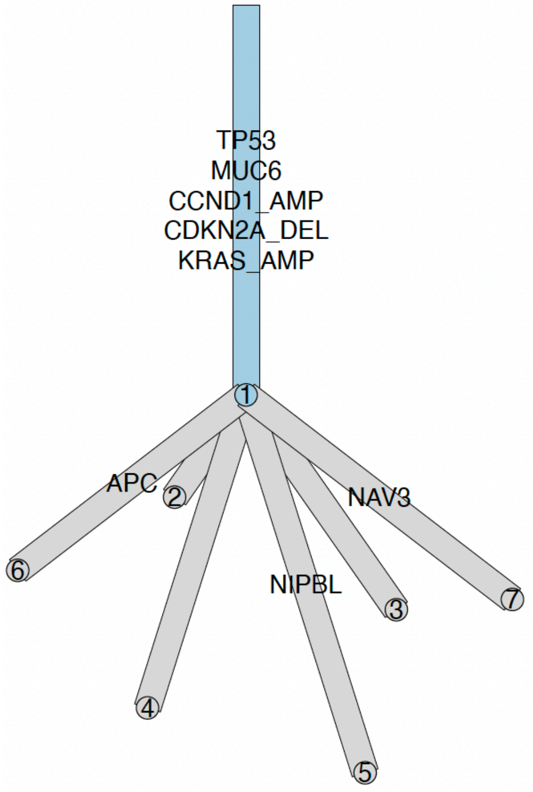
TP53
MUC6
CCND1_AMP
CDKN2A_DEL
KRAS_AMP
1
APC
2
NAV3
6
NIPBL
3
7
4
5

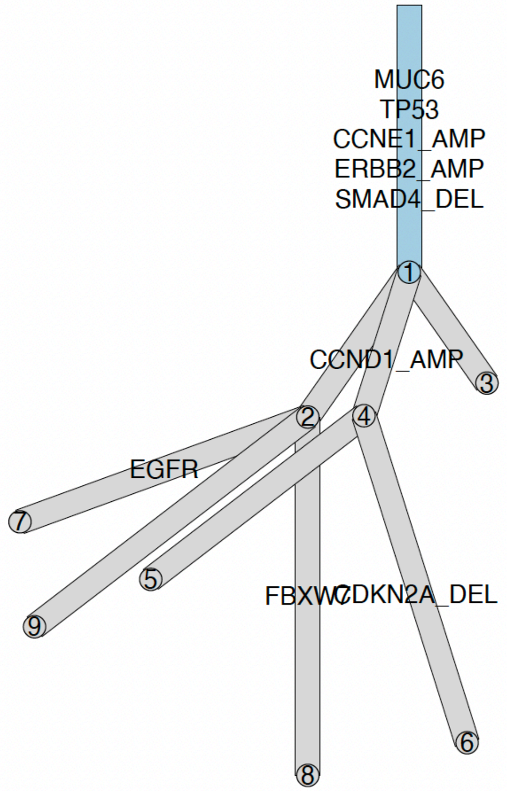
MUC6
TP53
CCNE1_AMP
ERBB2_AMP
SMAD4_DEL
1
CCND1_AMP
3
2
4
EGFR
7
5
FBXW7
CDKN2A_DEL
9
6
8

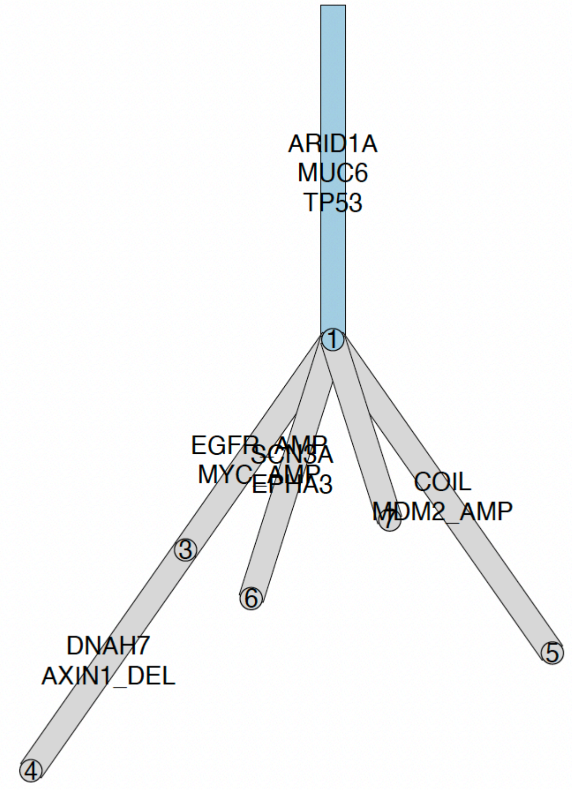
ARID1A
MUC6
TP53
1
EGFR_AMP
MYC_AMP
SCN3A
EPHA3
COIL
MDM2_AMP
7
3
6
5
DNAH7
AXIN1_DEL
4

**Cluster 7**

**BE+ve EAC**

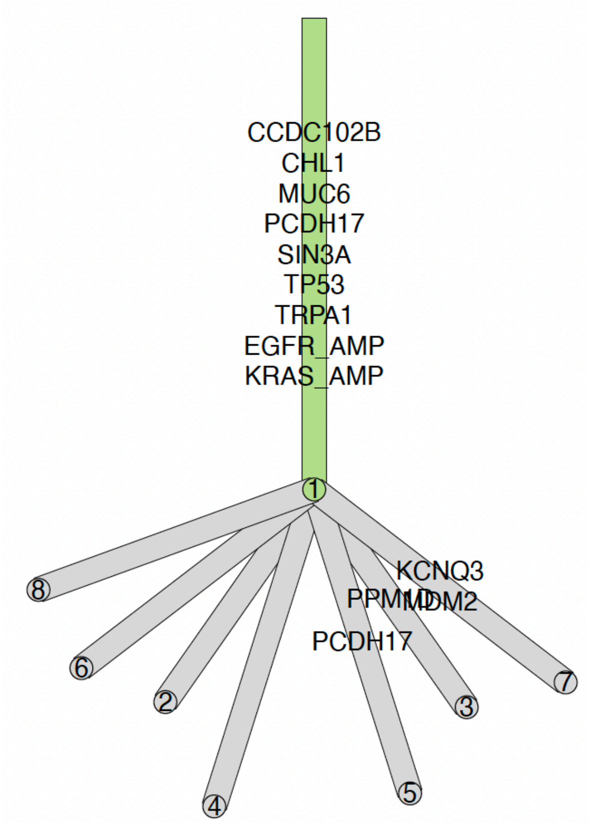


**BE-ve EAC**

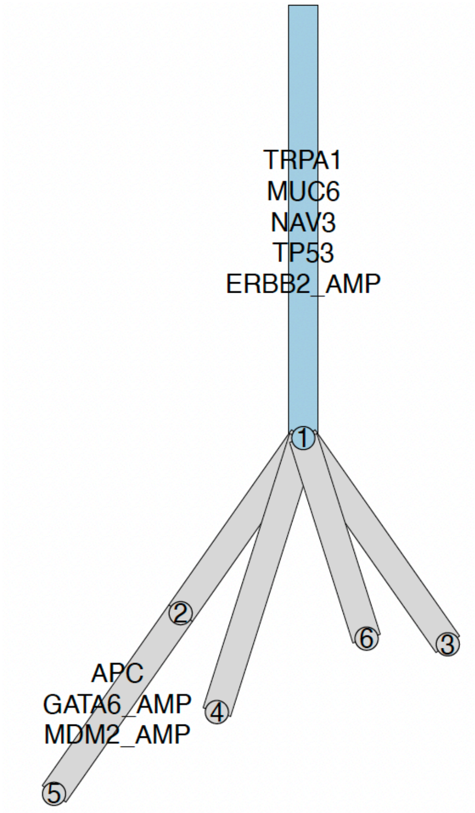

## Cluster 8

## BE+ve EAC

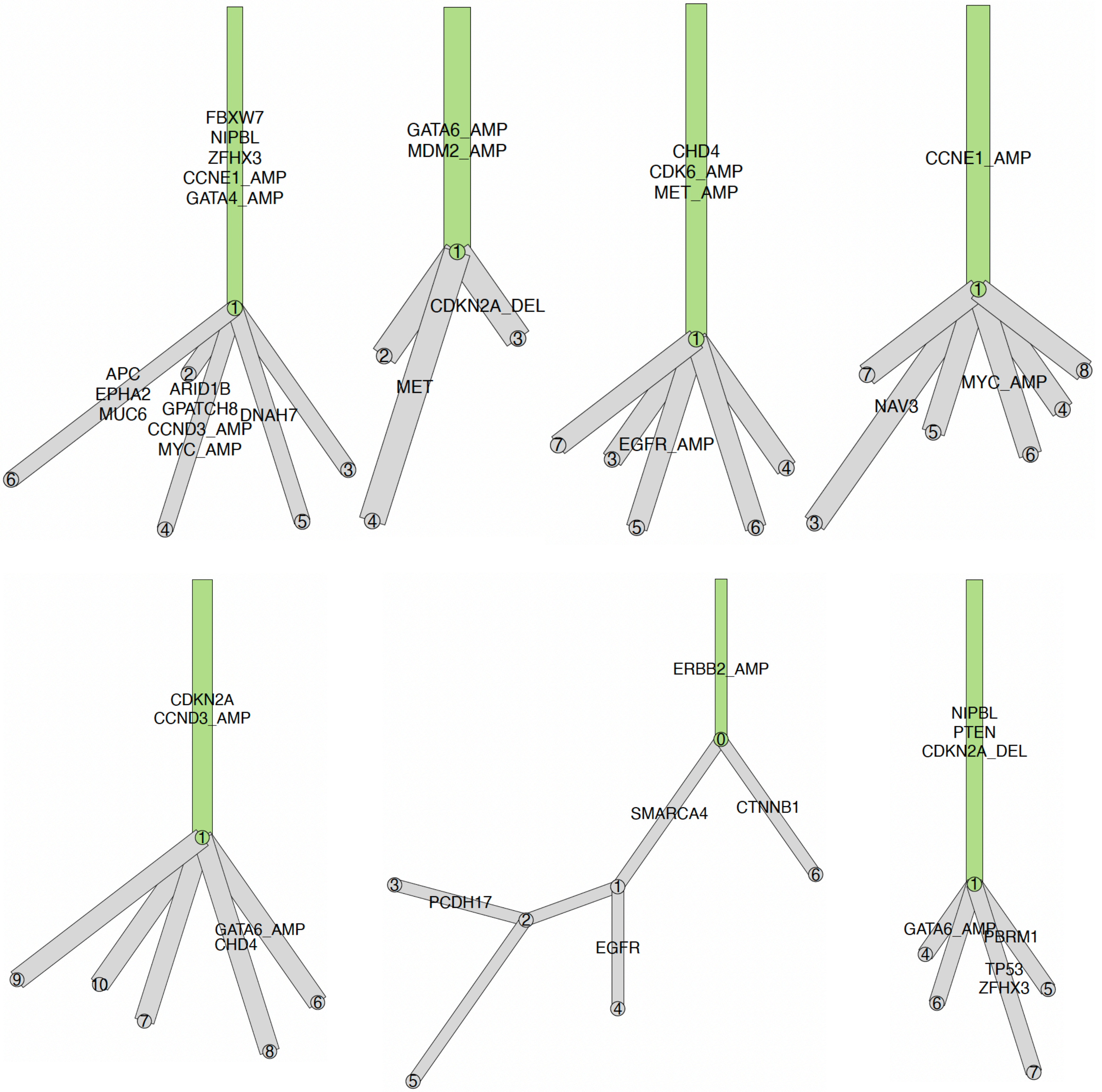

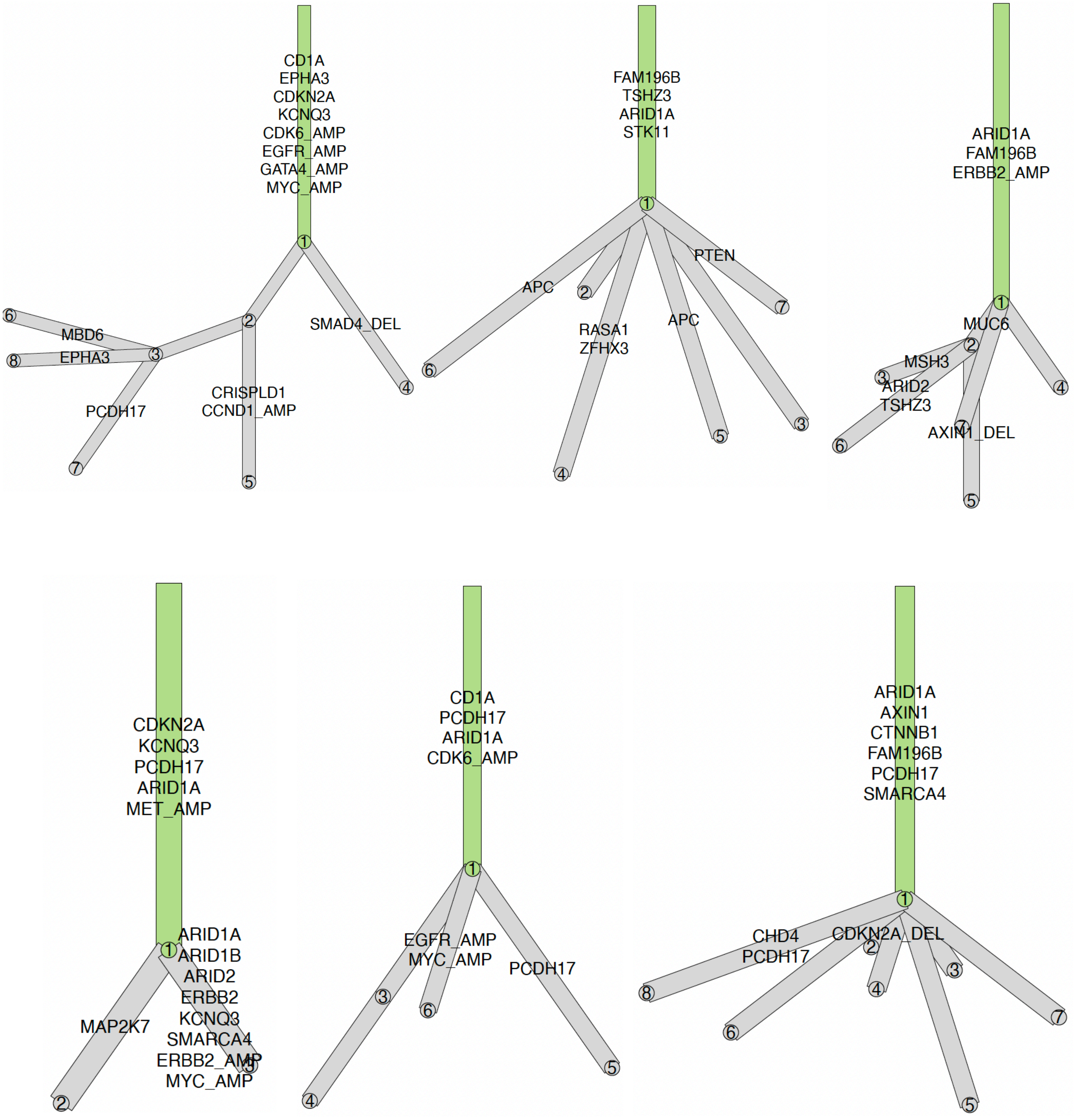
CD1A
EPHA3
CDKN2A
KCNQ3
CDK6_AMP
EGFR_AMP
GATA4_AMP
MYC_AMP
1
2
3
4
5
6
7
8
MBD6
EPHA3
PCDH17
CRISPLD1
CCND1_AMP
SMAD4_DEL
FAM196B
TSHZ3
ARID1A
STK11
1
2
3
4
5
6
7
APC
PTEN
RASA1
ZFHX3
APC
ARID1A
FAM196B
ERBB2_AMP
1
2
3
4
5
6
7
MUC6
MSH3
ARID2
TSHZ3
AXIN1_DEL
CDKN2A
KCNQ3
PCDH17
ARID1A
MET_AMP
1
2
3
ARID1A
ARID1B
ARID2
ERBB2
KCNQ3
SMARCA4
ERBB2_AMP
MYC_AMP
MAP2K7
CD1A
PCDH17
ARID1A
CDK6_AMP
1
3
4
5
6
EGFR_AMP
MYC_AMP
PCDH17
ARID1A
AXIN1
CTNNB1
FAM196B
PCDH17
SMARCA4
1
2
3
4
5
6
7
8
CHD4
PCDH17
CDKN2A_DEL

# BE-ve EAC

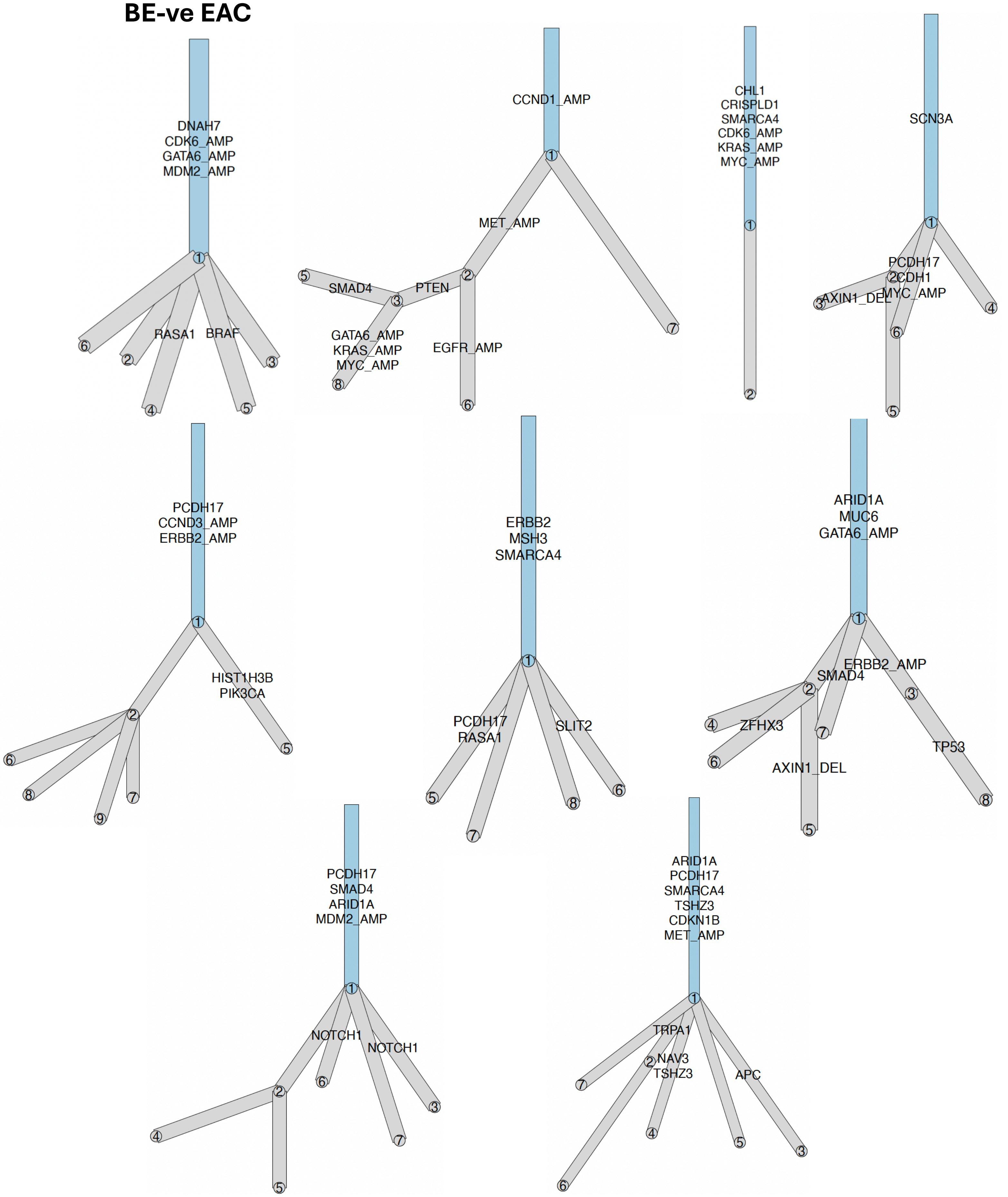
DNAH7
CDK6_AMP
GATA6_AMP
MDM2_AMP
RASA1
BRAF
CCND1_AMP
MET_AMP
SMAD4
PTEN
GATA6_AMP
KRAS_AMP
MYC_AMP
EGFR_AMP
CHL1
CRISPLD1
SMARCA4
CDK6_AMP
KRAS_AMP
MYC_AMP
SCN3A
PCDH17
CDH1
MYC_AMP
AXIN1_DEL
PCDH17
CCND3_AMP
ERBB2_AMP
HIST1H3B
PIK3CA
ERBB2
MSH3
SMARCA4
PCDH17
RASA1
SLIT2
ARID1A
MUC6
GATA6_AMP
ERBB2_AMP
SMAD4
ZFHX3
AXIN1_DEL
TP53
PCDH17
SMAD4
ARID1A
MDM2_AMP
NOTCH1
NOTCH1
ARID1A
PCDH17
SMARCA4
TSHZ3
CDKN1B
MET_AMP
TRPA1
NAV3
TSHZ3
APC
